# In vivo multiphoton microscopy detects longitudinal metabolic changes associated with delayed skin wound healing

**DOI:** 10.1038/s42003-018-0206-4

**Published:** 2018-11-19

**Authors:** Jake D. Jones, Hallie E. Ramser, Alan E. Woessner, Kyle P. Quinn

**Affiliations:** Department of Biomedical Engineering, University of Arkansas, 123 John A. White Jr. Engineering Hall, Fayetteville, AR 72701 USA

## Abstract

Chronic wounds are difficult to diagnose and characterize due to a lack of quantitative biomarkers. Label-free multiphoton microscopy has emerged as a useful imaging modality capable of quantifying changes in cellular metabolism using an optical redox ratio of FAD/(NADH+FAD) autofluorescence. However, the utility of an optical redox ratio for long-term in vivo monitoring of tissue metabolism has not been robustly evaluated. In this study, we demonstrate how multiphoton microscopy can be used to monitor changes in the metabolism of individual full-thickness skin wounds in vivo. 3D optical redox ratio maps and NADH fluorescence lifetime images identify differences between diabetic and control mice during the re-epithelialization of wounds. These metabolic changes are associated with a transient increase in keratinocyte proliferation at the wound edge. Our study demonstrates that high-resolution, non-invasive autofluorescence imaging can be performed in vivo and that optical redox ratios can serve as quantitative optical biomarkers of impaired wound healing.

## Abstract (web summary)

Jake Jones et al. show that changes in the metabolism of individual full-thickness skin wounds can be continuously measured in vivo using non-invasive autofluorescence imaging. This study presents endogenous optical biomarkers of chronic wounds, which offer promise in diagnosing impaired wound healing.

## Introduction

Chronic wounds are a major public health problem, affecting up to 2% of the total world population^1^, and costing ~25 billion dollars annually in the US^2^. Chronic wounds arise when the inflammatory and proliferative phases of skin wound healing become dysregulated due to poor vascularization, prolonged inflammation, callus formation, infection, or hyperglycemia^2–5^. Approximately 10–25% of patients suffering from diabetes mellitus will develop a non-healing foot ulcer, which is the most common reason for hospital admission of patients with the disease^6–8^. Current clinical approaches to diagnose and monitor foot ulcers, include symptomatic evaluation, wound size monitoring, and swab-based assays^9,10^. However, these non-invasive procedures provide very limited quantitative information in understanding wound pathogenesis. Histology and immunohistochemistry have provided key insights into the mechanisms of impaired healing and assisted in the development of advanced wound care products, but these techniques are inherently destructive and time-consuming. Therefore, there is a critical need to develop non-invasive quantitative biomarkers of wound healing to supplement current clinical management and guide product development.

Multiphoton microscopy (MPM) is well-suited for visualizing tissue in three dimensions at the cellular level^11–14^. Through the simultaneous absorption of two or more infrared photons, MPM provides intrinsic depth-sectioning, allows for increased imaging depths of more than 0.5 mm, and has minimal photodamage compared to confocal microscopy^15,16^. MPM can also be used to excite the naturally fluorescent electron carriers nicotinamide dinucleotide (NADH) and flavin adenine dinucleotide (FAD), which have a ubiquitous presence in cell metabolism^17–19^. These cofactors undergo oxidation/reduction reactions during glycolysis, the tricarboxylic acid (TCA) cycle, and oxidative phosphorylation. However, NADH is only fluorescent in the reduced form and FAD only fluoresces while oxidized^20,21^. An optical redox ratio of FAD/(NADH+FAD) fluorescence has been used in a variety of biomedical research applications and correlates with the intracellular concentrations of NAD^+^ and NADH^22–24^. Decreases in the optical redox ratio of cells or tissues have been attributed to hypoxia, the proliferative demands of cancer, and increased macromolecule biosynthesis^20,25^. We have also recently identified differences in the redox ratio between frozen tissue sections of diabetic and nondiabetic wounds^26^. However, very few studies have utilized an optical redox ratio to monitor metabolic changes in vivo in part due to the putative presence of other fluorophores or chromophores that can interfere with this ratiometric measurement^27^. While the use of an optical redox ratio has been primarily limited to in vitro or ex vivo applications, NADH fluorescence lifetime imaging (FLIM) has emerged as a viable method for in vivo metabolic assessments^13,28–31^. FLIM is intensity independent and measures the time that a molecule spends in an excited state before emission. The lifetime of NADH autofluorescence is highly sensitive to the fraction of free and protein-bound NADH^32–34^, and studies have demonstrated a sensitivity to hypoxia, proliferation, and biosynthesis similar to that of an optical redox ratio^35,36^. However, long acquisition times, high implementation cost, and the need for greater signal-to-noise have limited in vivo FLIM applications in dermatology and its clinical translation.

The goal of this study was to establish whether NADH and FAD autofluorescence could be used to non-invasively monitor wound healing dynamics in vivo over time and determine whether an optical redox ratio can serve as a quantitative biomarker of impaired wound healing. To this end, we employed high-speed volumetric imaging and image processing to generate 3D maps of metabolism within full-thickness, excisional wounds of diabetic and non-diabetic mice over 10 days. Changes in the optical redox ratio and NADH fluorescence lifetime demonstrated sensitivity to keratinocyte function at the wound edge and altered metabolism in diabetic wounds. To our knowledge, this study is the first successful use of an optical redox ratio to longitudinally monitor tissue metabolism in live animals over multiple days. The successful application of this method to in vivo skin wounds demonstrates that NADH and FAD autofluorescence offers promise in detecting keratinocyte dysfunction within chronic wounds.

## Results

### In vivo label-free MPM can monitor skin wounds over time

**Fig. 1:**
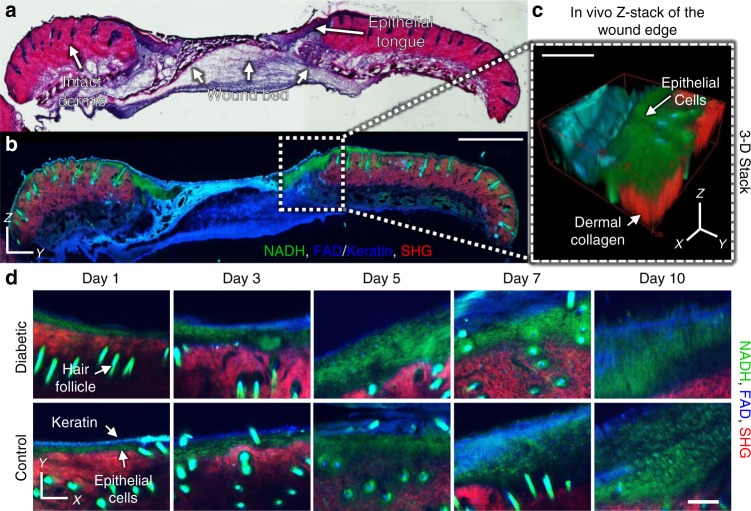
Comparison of in vivo MPM imaging at the wound edge with ex vivo tissue sections. **a** Hematoxylin and eosin stained tissue sections at 5 days post-wounding reveal distinct skin wound regions such as the dermis, epithelial tongue, and granulation tissue. **b** These same regions are visible through MPM imaging (scale bar = 1000 µm) of an adjacent unstained section, which highlights NADH (755 nm ex./460 nm em., green), FAD and keratin (900 nm ex./525 nm em., blue), as well as collagen SHG (900 nm ex./460 nm em., red). **c** Similar patterns of autofluorescence were detectable in the skin during in vivo acquisition of MPM z-stacks at the wound edge (scale bar = 200 µm). **d** Depth-resolved optical sections taken 125 µm deep into the wound edge demonstrate an ability to monitor the wound edge at all in vivo time points (scale bar = 100 µm)

To evaluate whether MPM could be used to assess differences in wound healing in vivo, streptozotocin-induced diabetic and control C57BL/6J mice were imaged longitudinally over a 10-day period following application of a full thickness, excisional wound on their dorsum. MPM image z-stacks were collected in vivo from locations at the wound edge that spanned from the surface of the epithelium down 250 µm into the dermis and wound bed (Fig. 1). Through image processing approaches to eliminate motion artifacts (Supplementary Fig. 1), high-contrast image stacks of NADH two-photon excited fluorescence (TPEF), FAD/keratin TPEF, and collagen SHG were produced, allowing visualization of individual cells in vivo (Fig. 1d; Supplementary Movie 1). In vivo MPM imaging also allowed for the easy delineation of tissue regions, such as the epidermis, dermis, and wound bed, similar to that of H&E stained wound sections (Fig. 1). The epidermis was identifiable by the presence of keratinocytes at the wound edge that produced strong emission at 460 nm using 755 nm excitation, which corresponds to NADH fluorescence (Fig. 1d; green). Within the stratum corneum, keratin autofluorescence was also observed with strong 525 nm emission at 900 nm excitation (Fig. 1d; blue). Beneath the epithelium, the uninjured dermis was recognizable by a strong SHG signal from type I collagen fibers (Fig. 1d; red). The presence of hair follicles was also apparent in the uninjured dermis adjacent to the wounds (Fig. 1). Hair follicles displayed a strong NADH autofluorescence and lacked any SHG signal, while the hair itself was intensely autofluorescent in all emission channels. The wound bed generally lacked any strong fluorescence or SHG signal during days 1–3 post-wounding before granulation tissue formed. At later time points, collagen SHG could be observed in the wound bed, but the normalized signal intensity was an order of magnitude lower than the adjacent dermis.

### Optical redox ratio detects differences in diabetic healing

**Fig. 2:**
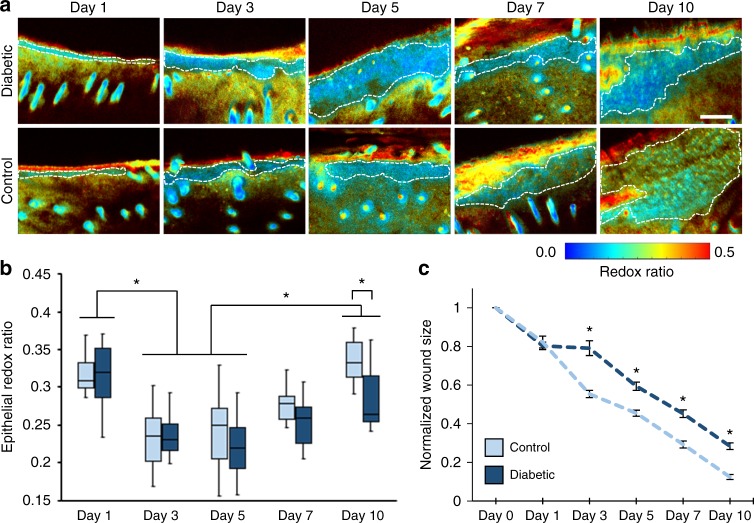
Optical redox ratio of the wound edge changes over time. **a** In vivo redox ratio maps of FAD/(NADH+FAD) were generated from the normalized fluorescence intensities. The representative optical sections from the z-stacks were acquired at the wound edge, 125 µm from the surface of the tissue (scale bar = 100 µm). An average optical redox ratio at the wound edge was calculated from the pixels contained within the epithelium (white dashed lines). **b** Both diabetic and control mice experienced an initial decrease in the epithelial redox ratio from day 1 to days 3 and 5 (*p* < 0.0002), followed by a gradual increase in redox ratio until day 10. By day 10, the diabetic group had a lower redox ratio than the controls (*p* = 0.0385). **c** Wound size in the diabetic group displayed characteristic delays in healing (*p* < 0.0001). Boxes correspond to the first and third quartiles, and whiskers extend to the full range of the data

With the ability to discriminate specific regions within the wound (Fig. 1), the optical redox ratio of FAD/(NADH+FAD) within the epithelial tongue could be spatially isolated and computed without interference from collagen or keratin^23^ (Fig. 2). Quantification of an average optical redox ratio within the epithelial tongue during longitudinal monitoring revealed temporal changes in metabolism (Fig. 2a). The redox ratio initially decreased in both the diabetic and control mice with lower values on day 3 (*p* < 0.0001) and day 5 (*p* < 0.0002) relative to day 1. By day 7, the redox ratio of individual wounds typically began to increase. At day 10, the mean redox ratio was greater than days 3 and 5 for both diabetic (*p* < 0.0441) and control mice (*p* < 0.0001). However, by day 10, diabetic mice had a lower epithelial redox ratio compared to controls (*p* = 0.0385), suggesting a metabolic sensitivity to a delay in wound closure (*p* < 0.0001; Fig. 2c).

### Epithelial redox ratio changes relate to cell proliferation

**Fig. 3:**
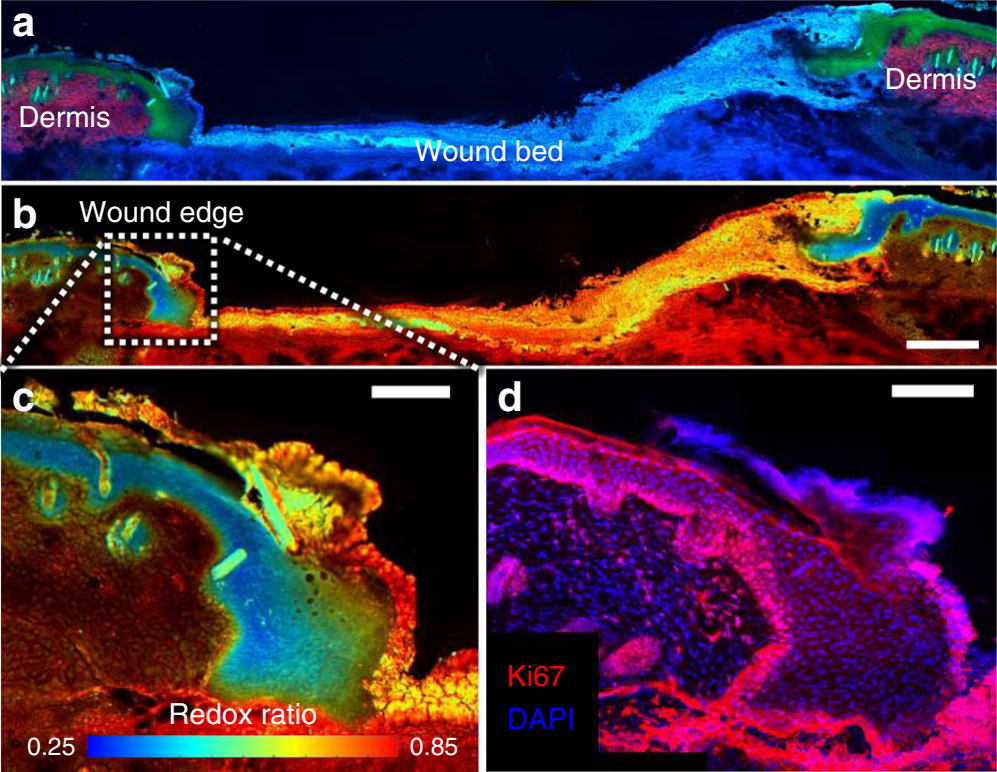
Spatial patterns in the optical redox ratio of the epithelial tongue indicate a sensitivity to proliferation. **a** Keratinocytes in the epithelial tongue of unstained wound sections, distinct from dermal collagen SHG and granulation tissue, produced a low optical redox ratio (scale bar = 250 µm). **b** A redox ratio gradient was observed in the epithelial tongue at the wound edge (scale bar = 100 µm). **c** Keratinocytes at the base of the tongue, proximal to the dermis, had lower redox ratio values than the keratinocytes at the tip toward the wound center. Ki67 immunostaining revealed a high density of proliferative keratinocytes at the base of the epithelial tongue corresponding to regions with a lower redox ratio (scale bar = 100 µm). **d** The tip of the epithelial tongue, displaying a higher redox ratio, contained no signs of proliferation

To elucidate the underlying cause of the observed changes in cell metabolism during longitudinal MPM monitoring, the spatial distribution of the optical redox ratio within the epithelial tongue was assessed in unstained ex vivo sections of wounds at different time points (*n* = 4 per time point). Wound section images were collected and normalized in the same manner as in vivo stacks (Fig. 3a), and ex vivo optical redox ratio maps were generated for entire wound cross sections (Fig. 3b). Interestingly, the ex vivo epithelial redox ratio was found to be higher (*p* < 0.001) than the redox ratio from corresponding in vivo image stacks. However, a strong correlation (*p* < 0.0001, *R* = 0.8655) between the ratios was found, indicating that the observed metabolic trends had been preserved (Supplementary Fig. 2). The redox ratios of keratinocytes in the ex vivo sections exhibited a distinct spatial gradient in the epithelial tongue (Fig. 3c) in which the cells at the base of the epithelium most proximal to the dermis had a lower redox ratio (~0.35) than the cells at the tip (~0.6) toward the wound center (Fig. 3c). These spatial patterns observed in ex vivo sections and temporal patterns observed during in vivo monitoring are consistent with the transition of keratinocytes from proliferating basal cells at the wound edge to migration into the wound bed^37^.

**Fig. 4:**
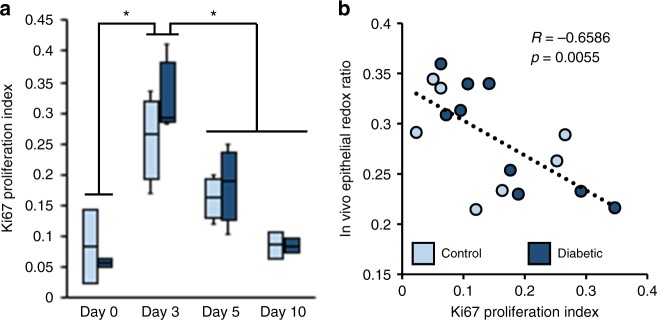
A quantitative proliferation index is correlated with in vivo optical redox ratio measurements. **a** A proliferation index measuring the ratio of DAPI-stained cells expressing Ki67 in the epithelial tongue revealed differences across time points (*p* < 0.02). Boxes correspond to the first and third quartiles, and whiskers extend to the full range of the data. **b** Proliferation index correlates with the mean in vivo redox ratio of the epithelial tongue

To evaluate the relationship between cell proliferation and the optical redox ratio in the epithelial tongue, adjacent histological sections were immunolabeled with Ki67. A spatial pattern similar to the redox ratio was observed (Fig. 3d), in which keratinocytes at the base of the epithelial tongue stained positively for Ki67, while the tip distinctly lacked any proliferative markers. Counterstaining with DAPI enabled calculation of a quantitative proliferation index, computed as the ratio of cells co-localizing DAPI and Ki67 (Ki67 ∩ DAPI) to the total number of DAPI expressing cells in the ex vivo epithelial tongue. The proliferation index, (Ki67 ∩ DAPI)/DAPI, of the histological sections (Fig. 4a) saw a >six-fold increase between days 0 and 3 in diabetic and control groups (*p* < 0.0183), followed by a decrease in proliferation in the subsequent days (*p* < 0.0200). The mean redox ratio of the epithelial tongue in adjacent tissue sections significantly correlated (*p* = 0.0235, *R* = −0.5797) with the proliferation index. A correlation was also observed between proliferation index and the redox ratio measured in vivo (*p* = 0.0055, *R* = −0.6586) (Fig. 4b). This data provides quantitative evidence that the change in optical redox ratio of the epithelial tongue is related to keratinocyte proliferation.

### NADH FLIM is sensitive to changes in proliferation

**Fig. 5:**
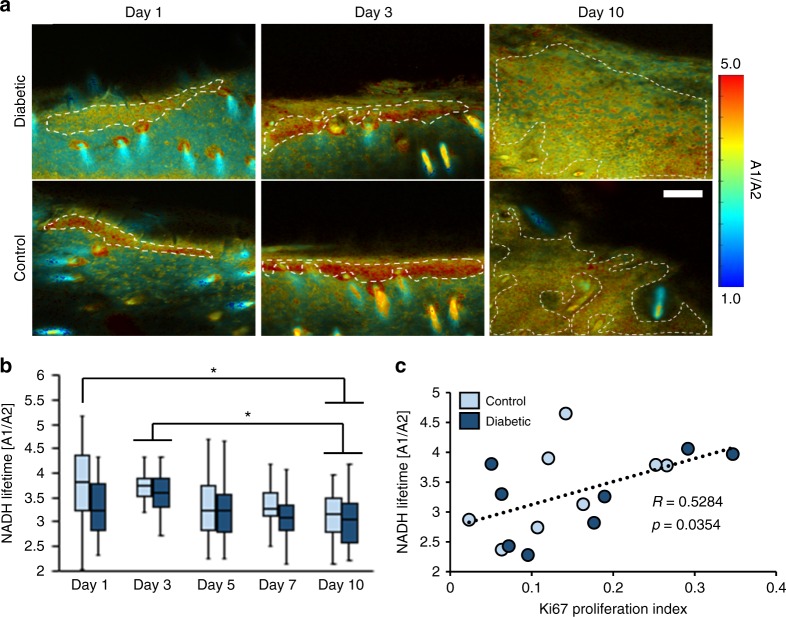
FLIM data from diabetic and control wound edges indicate changes in the ratio of free (A1) and protein-bound (A2) NADH lifetime components. **a** Representative maps of A1/A2 demonstrate changes in free-to-bound NADH ratios within the epithelium defined by the dashed lines (scale bar = 100 µm). **b** The average A1/A2 ratio within the epithelium was lower on day 10 compared to day 3 (*p* < 0.0095). Boxes correspond to the first and third quartiles, and whiskers extend to the full range of the data. **c** The mean A1/A2 ratio obtained in vivo was also significantly correlated with the Ki67 proliferation index (*p* = 0.0354)

NADH lifetime was used to further evaluate the dynamic changes in wound metabolism observed in vivo. Due to the long integration time (2 min) to acquire FLIM data through time-correlated single photon counting, a NADH lifetime image was acquired at the midpoint of each MPM z-stack in both diabetic and control mice. Phasor analysis of the FLIM data confirmed the presence of only two molecular species within the epithelial regions of the images (Supplementary Fig. 3). A bi-exponential fit of the fluorescence lifetime decay provided the relative contribution of free (A1) and protein-bound (A2) NADH at each pixel (Fig. 5a). Similar to the optical redox ratio, a ratio of A1/A2 was computed from the region where keratinocytes were present. Consistent lifetimes were measured for short (*τ*_1_ = 536 ± 88 ps) and long (*τ*_2_ = 3679 ± 302 ps) components, but both the diabetic and control mice displayed a lower A1/A2 ratio (*p* < 0.0095) on day 10 compared to their corresponding ratio values on day 3 (Fig. 5b). The decrease in A1/A2 observed over time after day 3 matched the temporal trend in proliferation rates (Fig. 4), and a correlation with the proliferation index was found (*p* = 0.0354, *R* =  0.5284; Fig. 5c). These FLIM results help to corroborate the observed changes in the optical redox ratio and further indicate an optical sensitivity to changes in keratinocyte proliferation during wound healing.

## Discussion

Although the autofluorescence of NADH and FAD has been measured in many biomedical applications over the past 70 years^17,21,25,27,32,38–40^, very few studies have demonstrated the use of an optical redox ratio in vivo^11,13,28,41^. By combining a method of fast MPM image acquisition with advanced post-processing to minimize motion artifacts and improve signal-to-noise ratios (Supplementary Fig. 1), we were able to monitor the healing process of individual wounds of streptozotocin-injected diabetic and control mice through 3D optical redox ratio maps at the wound edge (Figs. 1, 2). In vivo longitudinal studies have traditionally been challenging to perform due to the difficulty of isolating the contributions of the many endogenous fluorophores. Keratin, hair, fibrin clots, and SHG signal from dermal collagen are all detectable within the combination of excitation and emission wavelengths used for NADH and FAD imaging (Supplementary Movie 1). Furthermore, chromophores such as hemoglobin and melanin have presented challenges to measuring an optical redox ratio in vivo^24,42,43^. However, by focusing our analysis on keratinocytes within the edge of the wound, an optical redox ratio could be quantified that was free from the contribution of other chromophores. Phasor analysis of NADH lifetime confirmed the presence of only two molecular species corresponding to free and bound NADH within the epithelium (Supplementary Fig. 3), and bi-exponential fitting of NADH lifetime decay revealed a sensitivity to changes in metabolism that were consistent with in vivo optical redox ratio measurements (Fig. 5). By exploiting the high-resolution and high-throughput of 3D imaging at the wound edge, an optical redox ratio revealed distinct temporal trends in keratinocyte metabolism between diabetic and control mice (Fig. 2).

The transient decrease in redox ratio observed 3–5 days post-wounding was associated with the proliferation of keratinocytes within the epithelial tongue (Figs. 2–4). At the cellular level, proliferation requires a significant amount of biosynthesis to form the macromolecules necessary to replicate organelles, intracellular contents, and phospholipid membranes^44,45^. To meet these demands, cells increase their uptake of glucose and other carbon-rich molecules, and significantly more carbon is needed than ATP for the de novo synthesis of many macromolecules^44,46^. When glucose catabolism increases to accommodate biosynthesis without a proportional increase in oxidative phosphorylation, the intracellular concentration of NADH rises, causing a decrease in the optical redox ratio of FAD/(NADH+FAD) of proliferating cells. Studies using an optical redox ratio to monitor epithelial cancers have related accumulations of NADH to increased proliferative capacity^13,23,38,47^, while more metastatic carcinomas displayed a decrease in the concentration of NADH suggesting that invasive cells favor an oxidative metabolism^39,48,49^. These trends in the optical redox ratio between proliferative and invasive epithelial cancers match that of keratinocytes during wound re-epithelialization. The optical redox ratio decreases as keratinocytes proliferate at the wound edge during the early stages of healing, and then increases as cells migrate over the wound bed (Figs. 2, 3).

To further evaluate changes in keratinocyte metabolism during wound healing, fluorescence lifetime images of NADH were also acquired in vivo. Changes in NADH lifetime are typically attributed to changes in the free:bound ratio^34,50^. Through bi-exponential fits, this A1/A2 ratio of NADH lifetime species increases with glycolysis during the proliferation of epithelial cells^35^. FLIM studies of proliferative epidermal stem cells^35,51^ and carcinomas^13^ also demonstrate an accumulation of NADH and lower mean lifetimes. This may suggest a limited number of NADH protein-binding sites results in an accumulation of free NADH during epithelial cell proliferation. These studies are consistent with the decreased optical redox ratio and peak A1/A2 ratio measured at day 3 in the wound edge, as well as the significant correlation between the A1/A2 ratio and our proliferation index (Fig. 5). Interestingly, unlike optical redox ratio measurements made in vivo (Fig. 2) and ex vivo^26^, differences in NADH lifetime between the diabetic and control wounds were not observed at day 10 (Fig. 5). This may be related to the long acquisition times of time correlated single photon counting, which makes FLIM susceptible to motion-induced artifacts. Our image processing of the NADH and FAD intensity stacks minimized this problem and also allowed acquisition of metabolic data across multiple depths. Future work is needed to evaluate whether the optical redox ratio and FLIM outcomes offer complementary measures of distinct metabolic pathways utilized by keratinocytes as suggested in previous autofluorescence studies^24,52^.

We have demonstrated that MPM is capable of providing in vivo quantitative, longitudinal measurements of cell metabolism that are sensitive to the impaired healing process in diabetic wounds (Figs. 2, 5). Previous work has demonstrated that NADH fluorescence lifetime measurements can be used to assess tissue metabolism in vivo for a variety of applications^53–57^, but few studies have successfully demonstrated the utility of an in vivo optical redox ratio due to the putative effects of additional chromophores and the current limitations of clinical MPM systems, such as the MPTFlex^11,41^. While Skala et al. (2007) showed differences in the redox ratio of normal and precancerous tissues, this is the first study to our knowledge that demonstrates changes in the optical redox ratio of tissue within live animals imaged through repeated imaging sessions. This is a critical step in demonstrating the diagnostic potential of MPM for clinical wound care. Previous studies have explored the potential of MPM to monitor skin healing^57–59^, but this study demonstrates that label-free MPM can discriminate impaired healing resulting from diabetes mellitus. Here we show our optical sensitivity to wound healing dynamics is strongly associated with the demands of keratinocyte proliferation (Figs. 3, 4). Optical redox ratio and NADH lifetime sensitivity to the biosynthetic demands of the wound re-epithelialization are consistent with previous studies of epithelial tissue^41,60,61^. While assessments of epithelial cell heterogeneity were not quantified in this study, immune cells and other cell subpopulations within the epithelium may have distinct optical signatures, which can be quantified in future studies to understand their role in the healing process. A number of studies have also explored the utility of collagen SHG to provide structural information in dermatology applications. The ability of MPM to non-invasively collect both depth-resolved structural and metabolic data in vivo suggests MPM may have broader applicability for wound care and dermatology, including assessments of scarring. In the development of new wound therapies, these non-invasive imaging methods can provide quantitative biomarkers that can complement traditional tissue characterization through histology and immunohistochemistry. In the clinic, in vivo MPM may provide quantitative metrics of keratinocyte function that can supplement the current standard of care and guide treatment options. The application of in vivo MPM detailed here demonstrates an optical redox ratio and FLIM can serve as quantitative metrics sensitive to relevant changes in keratinocyte function produced in diabetic wounds.

## Methods

### Animal model of wound healing

All experiments were approved and performed according to the University of Arkansas IACUC (Protocol #16001). C57BL/6J mice (6 weeks; male) were divided into two groups and received daily intraperitoneal injections of either 50 mg/kg streptozotocin (*n* = 19) or buffer control (*n* = 18) for 5 days. Blood glucose was monitored in all mice with a ReliOn™ Blood Glucose monitor bi-weekly. Mice exhibiting blood glucose levels above 250 mg/dL after a 5-h fast were considered diabetic. Diabetic mice that were exhibiting a weight loss of 10% were given insulin at a dose of 0.1 units (if BG<350  mg/dL) or 0.2 units (if BG>350 mg/dL). At 12 weeks of age, the mice were anaesthetized for surgery with 2–5% isoflurane for induction and then maintained at 1–3%. On the day of wounding, the control group had a mean blood glucose of 129 ± 29 mg/dL while the diabetic group had a mean blood glucose of 454 ± 102 mg/dL. Mice were given carprofen (5 mg/kg, s.c.), and 6 mm full-thickness, excisional wounds were produced on the dorsum using a sterile biopsy punch. Excised tissue was flash frozen in Tissue-Tek^®^ optimal cutting temperature compound (Sakura Finetek; Tokyo, Japan) at −80 °C. Wound sizes were traced onto acetate paper at day 0 and at each imaging time point throughout the study to quantify closure. Tracings were digitized, and wound size was computed in MATLAB based on a normalized value relative to day 0. All wounds were bandaged with a primary covering of Tegaderm™ (3M; Maplewood, Maine) and secondary layer of surgical tape.

### In vivo multiphoton-excited fluorescence intensity imaging

Mice were placed under anesthesia and imaged on days 1, 3, 5, 7, and 10 post wounding for periods of no longer than 90 min. Three image stacks were collected at the wound edge (superior, inferior, and right lateral edge) per day using a Bruker Ultima Investigator laser scanning microscope (Middleton, Wisconsin) and Ti:sapphire laser (Spectra-Physics; Santa Clara, California). All images were acquired with a 20x, 1.0 NA water-immersion objective (Olympus; Tokyo, Japan). Fluorescence emission was collected using a 680 nm low-pass filter (Chroma, ET680sp-2p). A dichroic mirror at 495 nm (Chroma, T495lpxr) separated light into two GaAsP photomultiplier tubes (PMTs) (Hamamatsu; H10770PB-40). NADH autofluorescence was collected with one PMT using a 460 (±20) nm filter (Chroma, ET460/40m-2p)^40^ and 755 nm excitation. FAD autofluorescence was acquired at 900 nm excitation using the second PMT with a 525 (±25) nm emission filter (Chroma, ET525/50m-2p) to minimize contributions from NADH^62^. Second harmonic generation (SHG) signal was collected in the 460 nm channel at 900 nm. Z-stacks were acquired at three locations on the wound edge per animal consisting of images (512 × 512 pixels; 584 × 584 µm; 13-bit depth) taken en face from the top of the epithelium to a depth of 250 µm in increments of 5 µm. Rapid image acquisition (~70 ms per image slice; 3.5 s per stack) utilizing a piezo motor and 8 kHz resonant galvanometric scanning system was employed allowing for 50 sequential image stacks to be acquired in ~6–7 min for a given location. This high-speed acquisition limited motion artifacts to a small subset of the total acquired images, which could be removed later during image processing. Pixel dwell times during resonant scanning were 0.4 µs, and the cumulative pixel dwell time resulting from the acquisition of 50 z-stacks was 20 µs. Incident power never exceeded 50 mW at any depth.

### Image processing of 3D wound edge z-stacks

Fluorescence intensity images from all 50 image z-stacks were processed to remove images that contained motion artifact(s) and register stacks. The 50 intensity image stacks of the same excitation and location were averaged together with respect to depth to create an initial average intensity z-stack (Supplementary Fig. 1a). The individual images at a given depth in each of the 50 stacks were registered to the corresponding slice from the initial averaged stack using 2D cross correlation (Supplementary Fig. 1b). The cross-correlation maximums for each image were found and used to calculate a mean and standard deviation for the correlation at each depth in the 50 stacks. Any image with a cross-correlation value lower than one standard deviation of the mean correlation of the 50 images was then removed. An average of 7.7 ± 0.5% of the images were discarded per stack. The remaining images were registered and averaged together to form final high-contrast image stacks of NADH TPEF, FAD/keratin TPEF, and collagen SHG which enabled resolution of individual cells in vivo without mechanically restraining the tissue (Supplementary Fig. 1b). The final averaged image stacks of the 755 nm ex./460 nm em. channel (NADH TPEF) and 900 nm ex./525 nm em. channel (FAD/keratin TPEF) were registered together using a 3D cross-correlation algorithm and combined to create the final high-contrast in vivo wound edge stacks (Supplementary Fig. 1c; Supplementary Movie 1). Fluorescence intensities from the averaged stacks were normalized by laser power and PMT gain calibrated to µM concentrations of fluorescein in Tris buffer (pH 8) as described in previous studies^14,22^. Briefly, concentrations of fluorescein ranging from 0.1 µM to 20 µM were used to establish a power-law relationship between PMT voltage and power-normalized image intensity, allowing for corrections in any differences in PMT voltage across days. Laser power readings were acquired for normalization at every imaging time point to account for any day-to-day variability. Pixel-wise calculations of an optical redox ratio of [FAD/(NADH+FAD)] were computed using these normalized fluorescent intensities. Resulting redox ratio values were assigned to a jet color map in MATLAB for visualization (see Figs. 2, 3). The keratinocyte region within the stack was digitized using a manual tracing function in MATLAB to produce a mask of the epithelium based on the intensity image stack. The epithelial region was defined as the area between the stratum corneum and dermal collagen containing only keratinocytes. Autofluorescence from hair and hair follicles was avoided when tracing the region of interest by avoiding the region surrounding hair fluorescence within a 5–10 pixel radius. The average epithelial redox ratio for each z-stack at the wound edge was calculated from the redox ratio values within the traced regions of interest in the images at 1/4, 1/2, and 3/4 total stack depth.

### In vivo FLIM

NADH fluorescence lifetime data was collected at a depth corresponding to the midpoint of each image stack. Images were collected with a pixel dwell time of 4.8 µs using a standard (non-resonant) galvanometric scanning system. Integration time for the lifetime images was set to 2 min. Time-resolved data were processed using SPCImage 6.4 (Becker & Hickl Gmbh; Berlin, Germany). For processing, an instrument response function for the system was measured using the second harmonic signal of urea crystals. The full width at half maximum of the instrument response function was 0.25 ns. FLIM images were spatially binned twice to get total pixel photon counts of at least 10,000 in the pixels within the epithelial edge, and an incomplete multi-exponential model was chosen. Fits were generated using the measured instrument response function and a bi-exponential decay model to separate the long (*A*2) and short (*A*1) lifetime components of bound and free NADH, respectively^13,34^. Images with *χ*² values of <1.5 were considered to be appropriately fit. The epithelial region of interest was defined in the same manner as redox ratio calculations, in which intensity images were used to manually trace the epithelial region containing cells between the stratum corneum and dermal collagen, while avoiding signal from hair follicles. The mean *A*1/*A*2 ratio within the epithelium was calculated by averaging the ratios within the traced keratinocyte mask. Phasor plots were generated from the raw time-resolved data at a frequency of 80 MHz after deconvolving the instrument response function from the lifetime data as described by Martelo and colleagues^63^.

### Wound section processing and data collection

Approximately 1 cm^2^ of skin wound tissue was excised from the mice following euthanasia. Two diabetic and two control mice were euthanized after imaging on days 3 and 5 to provide tissue samples, while all other samples were harvested after day 10 imaging. All tissue samples were flash frozen in OCT at −80 °C as described above. The frozen wound tissue was sectioned in a Leica CM1860 cryostat (Wetzlar, Germany) into 30 µm-thick samples, transferred to glass slides, and stored at −80 °C. Samples were imaged for NADH, FAD, and SHG using the same filter sets described for in vivo imaging. The slides were then stained with hematoxylin and eosin (H&E) for histopathological evaluation. Adjacent slides in series were fixed with paraformaldehyde and fluorescently labeled using polyclonal Ki67 Rabbit IgG at 2.5 µg/mL and Alexa 488 tagged Goat Anti-Rabbit IgG (ThermoFisher Scientific; Waltham, Massachusetts) before being counterstained with 300 nM DAPI (ThermoFisher Scientific). A Ki67 proliferation index was calculated using a custom Matlab function. Briefly, this function required the epithelial tongue to be manually traced within the images. Traced regions were thresholded using Otsu’s method to produce a mask of Ki67-stained nuclei and a mask of DAPI stained nuclei. The resulting masks were overlaid and the cells where Ki67 and DAPI were co-localized (Ki67 ∩ DAPI) were counted. This value was normalized to the total count of DAPI expressing nuclei using a ratio of [(Ki67 ∩ DAPI)/DAPI].

### Statistical analysis

Changes in in vivo redox ratio, *A*1/*A*2 ratio, proliferation index, and wound size were assessed using two-factor ANOVAs with interactions to test for significant differences. The ANOVA design considered individual image locations as random effects nested with each mouse. Tukey’s HSD tests were used for all post hoc analysis. Comparisons with *p* < 0.05 were considered to be statistically significant. For correlations between measurements, significance was determined using a Pearson correlation coefficient assuming a null hypothesis that *R* = 0. Standard error (shown as error bars in Fig. 2) was calculated from the variance among mean values computed for individual animals. All statistical analysis was completed using JMP^®^ Pro 13 (Carry, NC).

### Code availability

The MATLAB code used to process in vivo multiphoton images in this study has been deposited into the public repository GitHub at https://github.com/kylepquinn/Code-and-Example-Data/.

## Electronic supplementary material

Supplementary Information

Supplementary Movie 1

Description of supplementary movie

## Data Availability

The data that support the findings of this study are available from the corresponding author upon reasonable request.

## References

[1] Gottrup F (2004). A specialized wound-healing center concept: importance of a multidisciplinary department structure and surgical treatment facilities in the treatment of chronic wounds. Am. J. Surg..

[2] Sen C (2009). Human skin wounds: a major and snowballing threat to public health and the economy. Wound Repair Regen..

[3] Oyibo SO (2001). The effects of ulcer size and site, patient's age, sex and type and duration of diabetes on the outcome of diabetic foot ulcers. Diabet. Med..

[4] Oyibo SO (2001). A comparison of two diabetic foot ulcer classification systems: the Wagner and the University of Texas wound classification systems. Diabetes Care.

[5] Singh N, Armstrong DG, Lipsky BA (2005). Preventing foot ulcers in patients with diabetes. JAMA.

[6] Frykberg R (2001). Diabetic foot disorders: a clinical practice guideline. Wounds.

[7] Martins-Mendes D (2014). The independent contribution of diabetic foot ulcer on lower extremity amputation and mortality risk. J. Diabetes Complicat..

[8] Reiber G (1999). Causal pathways for incident lower-extremity ulcers in patients with diabetes from two settings. Diabetes Care.

[9] Gardner SE, Frantz RA, Doebbeling BN (2001). The validity of the clinical signs and symptoms used to identify localized chronic wound infection. Wound Repair Regen..

[10] Moore K, McCallion R, Searle RJ, Stacey MC, Harding KG (2006). Prediction and monitoring the therapeutic response of chronic dermal wounds. Int. Wound J..

[11] Balu M (2013). In vivo multiphoton NADH fluorescence reveals depth-dependent keratinocyte metabolism in human skin. Biophys. J..

[12] Kretschmer S (2016). Autofluorescence multiphoton microscopy for visualization of tissue morphology and cellular dynamics in murine and human airways. Lab. Invest..

[13] Skala MC (2007). In vivo multiphoton microscopy of NADH and FAD redox states, fluorescence lifetimes, and cellular morphology in precancerous epithelia. Proc. Natl Acad. Sci. USA.

[14] Quinn K (2012). Characterization of metabolic changes associated with the functional development of 3D engineered tissues by non-invasive, dynamic measurement of individual cell redox ratios. Biomaterials.

[15] Georgakoudi I, Quinn KP (2012). Optical imaging using endogenous contrast to assess metabolic state. Annu. Rev. Biomed. Eng..

[16] Denk W, Strickler JH, Webb WW (1990). Two-photon laser scanning fluorescence microscopy. Science.

[17] Chance B, Thorell B (1959). Localization and kinetics of reduced pyridine nucleotide in living cells by microfluorometry. J. Biol. Chem..

[18] Chance B, Schoener B, Oshino R, Itshak F, Nakase Y (1979). Oxidation-reduction ratio studies of mitochondria in freeze-trapped samples. NADH and flavoprotein fluorescence signals. J. Biol. Chem..

[19] Scholz R, Thurman RG, Williamson JR, Chance B, Bucher T (1969). Flavin and pyridine nucleotide oxidation-reduction changes in perfused rat liver. I. Anoxia and subcellular localization of fluorescent flavoproteins. J. Biol. Chem..

[20] Mayevsky A, Rogatsky GG (2007). Mitochondrial function in vivo evaluated by NADH fluorescence: from animal models to human studies. Am. J. Physiol. Cell. Physiol..

[21] Huang Shaohui, Heikal Ahmed A., Webb Watt W. (2002). Two-Photon Fluorescence Spectroscopy and Microscopy of NAD(P)H and Flavoprotein. Biophysical Journal.

[22] Quinn KP (2013). Quantitative metabolic imaging using endogenous fluorescence to detect stem cell differentiation. Sci. Rep..

[23] Varone A (2014). Endogenous two-photon fluorescence imaging elucidates metabolic changes related to enhanced glycolysis and glutamine consumption in precancerous epithelial tissues. Cancer Res..

[24] Kolenc, O. & Quinn, K. Evaluating cell metabolism through autofluorescence imaging of NAD(P)H and FAD. *Antioxid. Redox. Signal*. (2018). 10.1089/ars.2017.7451 10.1089/ars.2017.7451 PMC6352511 29268621

[25] Levitt Jonathan M., McLaughlin-Drubin Margaret E., Münger Karl, Georgakoudi Irene (2011). Automated Biochemical, Morphological, and Organizational Assessment of Precancerous Changes from Endogenous Two-Photon Fluorescence Images. PLoS ONE.

[26] Quinn K (2016). Diabetic wounds exhibit distinct microstructural and metabolic heterogeneity through label-free multiphoton microscopy. J. Invest. Dermatol..

[27] Wu Y, Qu J (2005). Two-photon autofluorescence spectroscopy and second-harmonic generation of epithelial tissue. Opt. Lett..

[28] Skala Melissa C., Riching Kristin M., Bird Damian K., Gendron-Fitzpatrick Annette, Eickhoff Jens, Eliceiri Kevin W., Keely Patricia J., Ramanujam Nirmala (2007). In vivo multiphoton fluorescence lifetime imaging of protein-bound and free nicotinamide adenine dinucleotide in normal and precancerous epithelia. Journal of Biomedical Optics.

[29] Yaseen MA (2013). In vivo imaging of cerebral energy metabolism with two-photon fluorescence lifetime microscopy of NADH. Biomed. Opt. Express.

[30] Sun Y (2009). Fluorescence lifetime imaging microscopy: in vivo application to diagnosis of oral carcinoma. Opt. Lett..

[31] Jo JA (2010). In vivo simultaneous morphological and biochemical optical imaging of oral epithelial cancer. Ieee. Trans. Biomed. Eng..

[32] Blacker, T. et al. Separating NADH and NADPH fluorescence in live cells and tissues using FLIM. *Nat. Commun*. **5**, 3936 (2014). 10.1038/ncomms4936 PMC4046109 24874098

[33] Yu Q, Heikal A (2009). Two-photon autofluorescence dynamics imaging reveals sensitivity of intracellular NADH concentration and conformation to cell physiology at the single-cell level. J. Photochem. Photobiol. B.

[34] Lakowicz JR, Szmacinski H, Nowaczyk K, Johnson ML (1992). Fluorescence lifetime imaging of free and protein-bound NADH. Proc. Natl Acad. Sci. USA.

[35] Stringari, C. et al. Metabolic trajectory of cellular differentiation in small intestine by Phasor Fluorescence Lifetime Microscopy of NADH. *Sci. Rep*. **2**, 568 (2012). 10.1038/srep00568 PMC3416911 22891156

[36] Stringari Chiara, Nourse Jamison L., Flanagan Lisa A., Gratton Enrico (2012). Phasor Fluorescence Lifetime Microscopy of Free and Protein-Bound NADH Reveals Neural Stem Cell Differentiation Potential. PLoS ONE.

[37] Park S (2017). Tissue-scale coordination of cellular behaviour promotes epidermal wound repair in live mice. Nat. Cell Biol..

[38] Walsh A (2013). Optical metabolic imaging identifies glycolytic levels, subtypes, and early-treatment response in breast cancer. Cancer Res..

[39] Alhallak K, Rebello L, Muldoon T, Quinn K, Rajaram N (2016). Optical redox ratio identifies metastatic potential-dependent changes in breast cancer cell metabolism. Biomed. Opt. Express.

[40] Rice WL, Kaplan DL, Georgakoudi I (2010). Two-photon microscopy for non-invasive, quantitative monitoring of stem cell differentiation. PLoS One.

[41] Dimitrow E (2009). Sensitivity and specificity of multiphoton laser tomography for in vivo and ex vivo diagnosis of malignant melanoma. J. Invest. Dermatol..

[42] Mansfield James R., Gossage Kirk W., Hoyt Clifford C., Levenson Richard M. (2005). Autofluorescence removal, multiplexing, and automated analysis methods for in-vivo fluorescence imaging. Journal of Biomedical Optics.

[43] Huang Zhiwei, Zeng Haishan, Hamzavi Iltefat, Alajlan Abdulmajeed, Tan Eileen, McLean David I., Lui Harvey (2006). Cutaneous melanin exhibiting fluorescence emission under near-infrared light excitation. Journal of Biomedical Optics.

[44] Vander Heiden MG, Cantley LC, Thompson CB (2009). Understanding the Warburg effect: the metabolic requirements of cell proliferation. Science.

[45] Lunt S, Vander Heiden M, Schekman R, Goldstein L, Lehmann R (2011). Aerobic glycolysis: meeting the metabolic requirements of cell proliferation. Annu. Rev. Cell Dev. Biol..

[46] Vander Heiden MG (2011). Metabolic pathway alterations that support cell proliferation. Cold Spring Harb. Symp. Quant. Biol..

[47] Ostrander JH (2010). Optical redox ratio differentiates breast cancer cell lines based on estrogen receptor status. Cancer Res..

[48] Li L (2009). Quantitative magnetic resonance and optical imaging biomarkers of melanoma metastatic potential. Proc. Natl.Acad. Sci. USA.

[49] Sun N, Xu HN, Luo Q, Li LZ (2016). Potential. Adv. Exp. Med. Biol..

[50] Berezin MY, Achilefu S (2010). Fluorescence lifetime measurements and biological imaging. Chem. Rev..

[51] Stringari C (2015). In vivo single-cell detection of metabolic oscillations in stem cells. Cell Rep..

[52] Liu Z (2018). Mapping metabolic changes by noninvasive, multiparametric, high-resolution imaging using endogenous contrast. Sci. Adv..

[53] Meleshina, A. et al. Two-photon FLIM of NAD(P)H and FAD in mesenchymal stem cells undergoing either osteogenic or chondrogenic differentiation. *Stem Cell Res. Ther.* **8**, 10.1186/s13287-017-0484-7 (2017). 10.1186/s13287-017-0484-7 PMC5273806 28129796

[54] Shah Amy T., Demory Beckler Michelle, Walsh Alex J., Jones William P., Pohlmann Paula R., Skala Melissa C. (2014). Optical Metabolic Imaging of Treatment Response in Human Head and Neck Squamous Cell Carcinoma. PLoS ONE.

[55] Stuntz, E. et al. Endogenous two-photon excited fluorescence imaging characterizes neuron and astrocyte metabolic responses to manganese toxicity. *Sci. Rep*. **7**, 10.1038/s41598-017-01015-9 (2017). 10.1038/s41598-017-01015-9 PMC5430620 28432298

[56] Hato T (2017). Two-photon intravital fluorescence lifetime imaging of the kidney reveals cell-type specific metabolic signatures. J. Am. Soc. Nephrol..

[57] Deka Gitanjal, Wu Wei-Wen, Kao Fu-Jen (2012). In vivowound healing diagnosis with second harmonic and fluorescence lifetime imaging. Journal of Biomedical Optics.

[58] Li J (2015). Effect of recombinant interleukin-12 on murine skin regeneration and cell dynamics using in vivo multimodal microscopy. Biomed. Opt. Express.

[59] König K (2007). Clinical two-photon microendoscopy. Microsc. Res. Tech..

[60] Balu M (2014). Distinguishing between benign and malignant melanocytic nevi by in vivo multiphoton microscopy. Cancer Res..

[61] Brem H (2007). Molecular markers in patients with chronic wounds to guide surgical debridement. Mol. Med..

[62] Zipfel WR (2003). Live tissue intrinsic emission microscopy using multiphoton-excited native fluorescence and second harmonic generation. Proc. Natl Acad. Sci. USA.

[63] Martelo L, Fedorov A, Berberan-Santos M (2015). Fluorescence phasor plots using time domain data: effect of the instrument response function. J. Phys. Chem. B.

