# Supplementary material for: In vivo multiphoton microscopy detects longitudinal metabolic changes associated with delayed skin wound healing
Source: Commun Biol. 2018 Nov 19;1:198. doi: 10.1038/s42003-018-0206-4 (PMC6242983; doi:10.1038/s42003-018-0206-4)
Supplement: Supplementary file 1 — Supplementary Information [file 42003_2018_206_MOESM1_ESM.pdf]

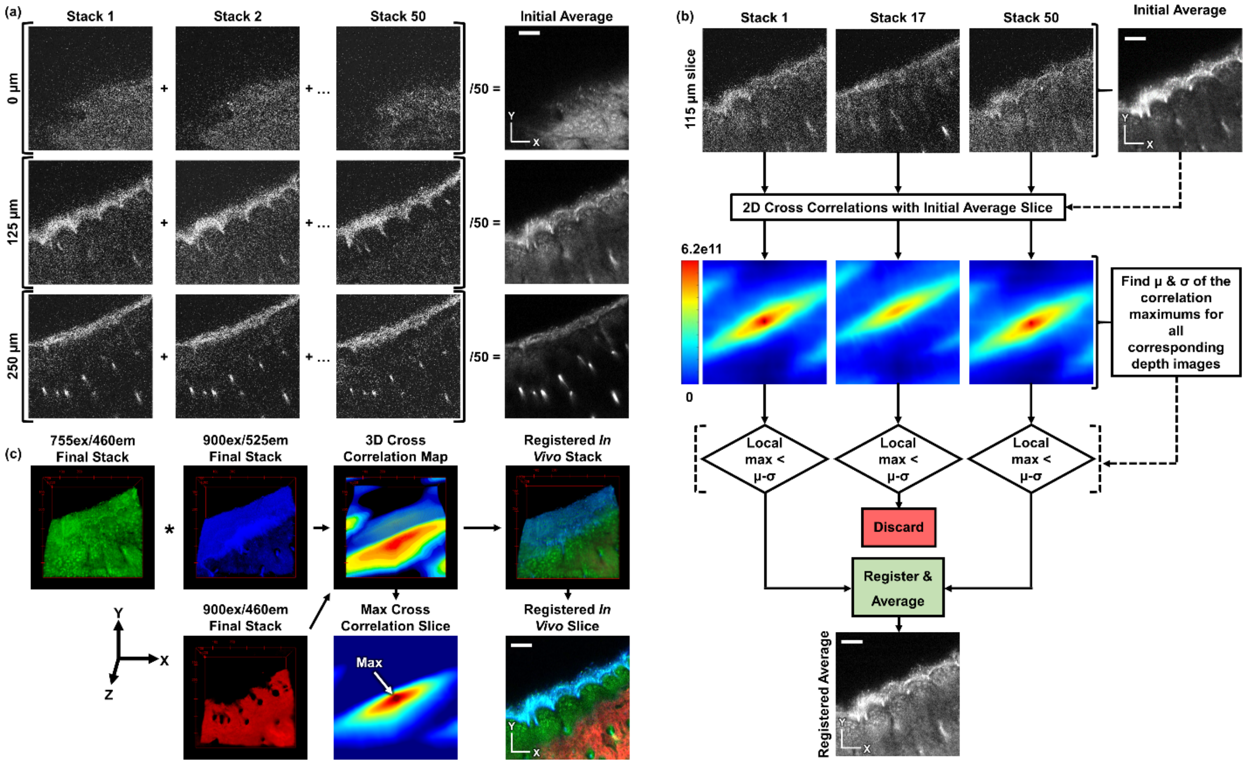

Supplementary Figure 1. Image processing of fluorescence intensity stacks minimized the effects of movement during in vivo imaging. (a) Fluorescence intensity images from all 50 image stacks of the same excitation and location were averaged with respect to depth to create an initial intensity average stack. (b) The individual image slices at a given depth in each of the 50 z-stacks were then registered to the corresponding averaged image slice using 2D cross correlation. The cross correlation maximums for each image were found, and used to calculate a mean and standard deviation for the maximum correlation for each depth in the 50 stacks. Any image with a maximum cross correlation value lower than one standard deviation of the average maximum was then removed. An average of 7.7±0.5% of acquired images per stack were misaligned and discarded due to breathing motion during in vivo acquisition. The remaining images were registered using the location of the maximum correlation and then averaged together to create a final registered average z-stack. (c) The final averaged image stacks of the 755nm ex./460nm em. channel (NADH) and 900nm ex./525nm em. channel (FAD) were then registered together using a 3D cross correlation algorithm and combined to create the final in vivo wound edge stacks. Second harmonic generation signal was registered using the same transform as FAD because the channels were acquired simultaneously. All scale bars shown are 100 µm.

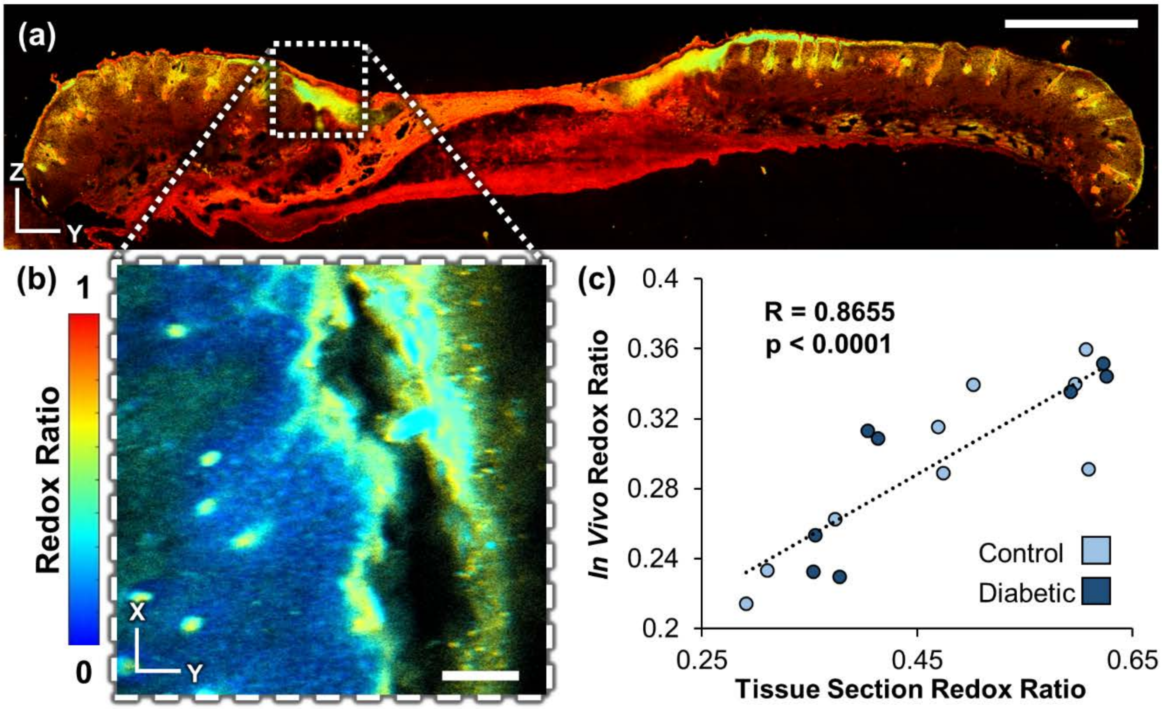

Supplementary Figure 2. Changes in redox ratio were observed between (a) ex vivo tissue sections and (b) in vivo image z-stacks. After in vivo image stacks (b) were acquired from the wound edge, the wound tissue was biopsied, frozen and sectioned (Scale bar 100 µm). These unstained sections (a) were imaged using the same excitation wavelengths and filter sets as the in vivo image stacks but displayed uniformly higher redox ratios (Scale bar 1000 µm). Despite this shift, the redox ratios of the ex vivo and in vivo epithelia were strongly correlated (c), suggesting a similar sensitivity to changes in keratinocyte metabolism.

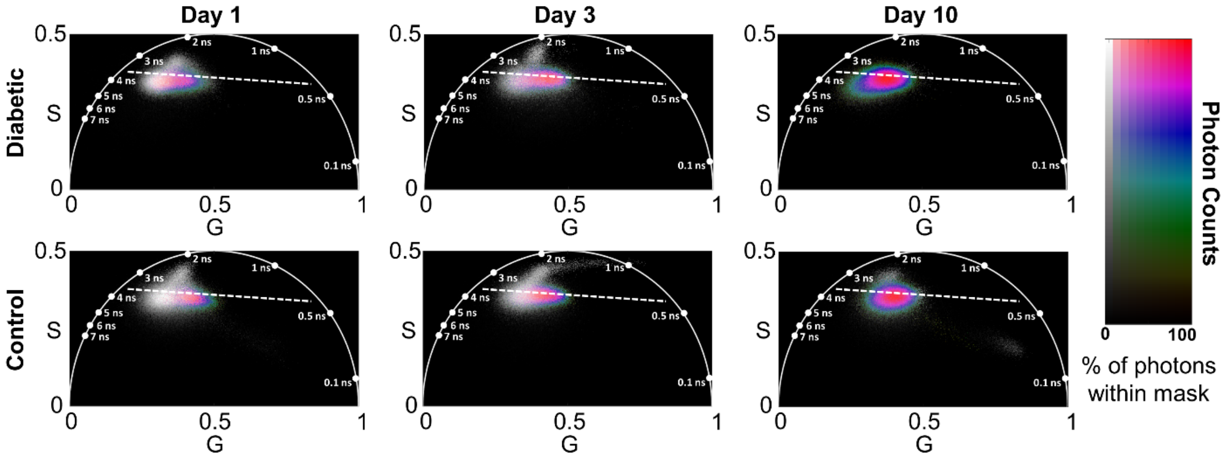

Supplementary Figure 3. Representative phasor plots (f = 80MHz) of NADH lifetime indicate the presence of two lifetime species within the epithelium. Phasor plots correspond to the representative images shown in Figure 5. Regions in color correspond to pixel locations within the epithelium (Fig. 5), while desaturated (grayscale) regions correspond to locations outside of the epithelial mask within the images. Phasor coordinates for the epithelial mask fall along the (white dashed) line corresponding to the average short (0.536ns) and long (3.679ns) components derived from biexponential fitting.
