# Supplementary material for: In vivo multiphoton microscopy detects longitudinal metabolic changes associated with delayed skin wound healing
Source: Commun Biol. 2018 Nov 19;1:198. doi: 10.1038/s42003-018-0206-4 (PMC6242983; doi:10.1038/s42003-018-0206-4)
Supplement: Supplementary file 3 — Description of supplementary movie [file 42003_2018_206_MOESM3_ESM.docx]

**Description of Additional Supplementary Files**

**File Name**: Supplementary Movie 1

**Description**: Example of an in vivo wound edge image z-stack spanning from the surface of the epithelium down 250 µm into the dermis and wound bed. The 3D stacks allow for individual keratinocytes (green, NADH) to be identified in the epithelium located in between collagen SHG (red) in the dermis and keratin autofluorescence from the stratum corneum (blue). Hair follicles displayed a strong NADH autofluorescence and lacked any SHG (red) or FAD (blue) signal, while the hair itself was intensely autofluorescent in all emission channels. These stacks allowed for keratinocyte fluorescence in the epithelial tongue to be isolated and quantitatively evaluated in the form of an optical redox ratio.
